# Technological and Safety Characterization of Coagulase-Negative Staphylococci Isolated from Sardinian Fermented Sausage Made by Ovine Meat

**DOI:** 10.3390/foods13040633

**Published:** 2024-02-19

**Authors:** Nicoletta P. Mangia, Michele Cottu, Maria Aponte, Marco A. Murgia, Maria E. Mura, Giuseppe Blaiotta

**Affiliations:** 1 Dipartimento di Agraria, University of Sassari, Viale Italia 39, 07100 Sassari, Italy; nmangia@uniss.it (N.P.M.); mamurgia@uniss.it (M.A.M.); mariaelenamura@uniss.it (M.E.M.); 2 Department of Agricultural Sciences, University of Naples Federico II, Via Università 100, 80055 Naples, Italy; blaiotta@unina.it

**Keywords:** coagulase-negative staphylococci, sheep fermented sausage, technological properties, proteolytic activity, SDS-PAGE, antibiotic resistance

## Abstract

Ripened sheep sausages are widely consumed in Italy, particularly in Sardinia. Despite their driving role in flavor and color development, coagulase-negative staphylococci in these products have been rarely investigated. A total of 70 CoNS cultures isolated from Sardinian sheep sausages were characterized by rep-PCR and M13-RAPD typing and identified by 16S rDNA sequencing. *S. xylosus* and *S. equorum* accounted for more than 70% of the total isolates, whilst *S. pasteuri* (8.5%), *S. succinus* (2.8%), and *S. haemolyticus* (2.8%) were less represented. The genes encoding the synthesis of putrescine, tyramine, cadaverine, and histamine were evaluated by PCR. None of the strains hosted genes for decarboxylases, except one *S. pasteuri* strain that was potentially a tyramine-producer. Antibiotic resistance was evaluated, along with nitrate reductase, lipolytic, and proteolytic activity, in a pool of selected cultures. Resistance to the primary antibiotics was rather widespread. *S. xylosus*, *S. equorum*, and *S. pasteuri* strains were all resistant to amoxicillin and kanamycin. *S. equorum* strains were sensitive to all tested antibiotics. *S. xylosus* strains were all resistant to penicillin B. Conversely, all *S. pasteuri* strains were resistant to both ampicillin and penicillin B, and four out of five strains exhibited tetracycline resistance. The high variability in the production of sheep sausages makes the search for adjunct cultures of crucial relevance. According to this perspective, the characterization of the autochthonous CSN population represents the first step to approach a starter selection.

## 1. Introduction

Ripened sheep sausage, which is essentially a type of salami, has a specific role among the Sardinian (Italy) stuffed meat-based fermented products. Its manufacturing is likely the result of the abundant supply of sheep meat from adult animals, especially from those that should not be consumed for public health reasons. Sardinia raises 42% of Italy’s sheep, and the region has the greatest national consumption of sheep meat. Sheep meat is suitable for making halal products, a market that is constantly changing in Italy [1]. The production technology is comparable to the method used to make pork sausages, but undoubtedly, more care is required for meat selection and preparation. For instance, fat and connective tissues, which may be responsible for unpleasant tastes, need to be carefully removed [1]. Furthermore, proper hygiene measures must be applied during animal slaughter, particularly in the skinning and evisceration phases, to prevent microbiological contamination of the carcass. 

The technological procedure utilized to produce sheep sausage was described in detail by Mangia et al. [2] and is summarized in Supplementary Figure S1. Briefly, sheep meat is minced and sometimes combined with pork fat in varying amounts based on consumers’ preferences. The meat mixture is left to set down overnight in a cool environment and stuffed into the pork/sheep casings. After that, the sausage is held for 20–30 days at 10–12 °C and 80% RU after being smoked for up to 4 days at 18 °C.

Except for Pitina, a typical fermented meat product of mountainous areas of North-East Italy whose origin dates back to the first half of the XIX century [3] in Italy, all these productions did not deserve too much room in the current scientific literature. Conversely, even though processed meat products from sheep and goats are not as popular as those from pork, beef, or poultry, there has been a noticeable growth in their production worldwide [4]. Young lambs and kids producing light carcasses are highly valued by consumers in various Mediterranean European countries; many of them are well-known PDO (Protected Designation Origin) or PGI (Protected Geographical Indication) brands [4]. The meat of sheep and goats that do not meet these quality marks is cured and processed to make well-known goods like fermented sausages that are either fresh or smoked. Sucuk, for instance, is a dry-fermented Turkish sausage widely consumed in Southeastern Europe, the Middle East, and Middle Asia [5], whilst Fjellmorr and Lambaspaeipylsa are dry-fermented sausages made in Norway and Iceland that contain lamb in addition to beef and pork [6]. Apart from these traditional productions, several studies investigated the incorporation of sheep or goat meat in sausages. The meat of sheep and goats was pointed out as a valuable alternative for producing sausages with different levels of pork fat [7]. Furthermore, fresh ewe sausages were found to be less fibrous and juicier than goat sausages based on sensory characterization and consumer preference mapping [8].

Because of the traditional technology, often inadequate and very diversified, it is difficult to find a product with consistent qualitative features. Most critically, product safety hinges on comprehensively understanding the factors influencing microbial presence and contamination throughout the production chain. Lactic Acid Bacteria (LAB), despite being dominant, showed a slow growth rate during the early stages of fermentation, thus allowing the coliforms’ survival [2]. The lactic microflora of Sardinian sheep sausages has been recently characterized, whilst no information is currently available concerning the staphylococcal populations [1].

Staphylococci include the coagulase-negative staphylococci (CoNS) group, which drives important biochemical reactions involved in the flavor and color development of fermented meat sausages [9]. Consequently, some species belonging to CoNS are often proposed as starter cultures, such as *Staphylococcus* (*S*.) *xylosus* and *S. carnosus* [10]. Proteolysis and lipolysis are the processes that are primarily in charge of the organoleptic and textural features of the products during the fermentation and ripening of sausages [11]. Feng et al. [12] demonstrated that proteolysis is catalyzed by not only endogenous enzymes but also proteases of microbial origin. Although endogenous enzymes are primarily responsible for protein degradation, bacterial proteases and peptidases play a significant role in the initial breakdown of myofibrillar and sarcoplasmic proteins, as well as in the release of small peptides and amino acids during the later stages of ripening [13]. Due to their nitrate reductase activity, which results in the synthesis of nitroso myoglobin, CoNS contribute to the development and stability of the product color, as well as to the limitation of lipid oxidation [14]. The development of flavor and texture in meat products is also greatly influenced by *Staphylococcus* species rather than LAB [11]. On the other hand, some staphylococci have the potential to form biofilms and exhibit hemolytic, DNase, and amino acid decarboxylase activity, which can influence the safety of products and pose a risk to human health [15]. According to various research [10], the prevalence of antibiotic resistance of CoNS from animal-origin foods is a growing issue for public health. Therefore, selecting starter cultures devoid of these activities is crucial. 

In this context, the aim of the present study is the isolation and characterization of staphylococci cultures previously isolated from Sardinian sheep sausages, with a focus on biochemical characteristics of technological interest, as well as the assessment of traits of safety relevance.

## 2. Materials and Methods

### 2.1. Microorganisms and Cultural Conditions

Seventy CoNS, previously isolated from ewe sausages traditionally made without starter cultures [2], were inoculated into Brain Heart Infusion (BHI) broth (Oxoid, Basingstoke, UK) and incubated at 37 °C for 24 h. Cultures were repeatedly streaked onto plates of the same medium to ensure purity and stored at −80 °C in BHI added of glycerol (20%).

### 2.2. Strains Identification and Genetic Characterization

Cultures in the exponential phase of growth were subject to DNA extraction by means of the ArchivePure DNA Yeast and Gram-+ Kit (5 PRIME GmbH, Hamburg, Germany), according to the manufacturer’s instructions. The 260/280 nm ratio (LVis Plate, SpectroNano system, BMGTech, Ortenberg, Germany) was used to evaluate the DNA quality. Prior to identification, strains were subject to a polyphasic approach to evaluate the intra-species variability. Randomly Amplified Polymorphic DNA-Polymerase Chain Reaction (RAPD-PCR) and Repetitive element palindromic–Polymerase Chain Reaction (rep-PCR), using the primers M13 (5′GAGGGTGGCGGTTCT-3) and GTG5 (5′-GTGGTGGTGGTGGTG-3), respectively, were carried out according to indications provided by Mangia et al. [1]. SYBR safe (Invitrogen, Waltham, MA, USA) was used for staining, and the Chemi Doc XRS imaging system (BioRad Laboratories, Milan, Italy) was used for visualization. Using the InfoQuest FP Software (version 4.5; Bio-Rad Laboratories, Hercules, CA, USA), cluster analysis of the patterns acquired from RAPD and rep-PCR analysis was carried out. Pearson’s correlation similarity coefficients were used to build a matrix of bacterial banding profiles’ similarities. An arbitrary cluster cut-off value of 85% was used to discriminate different bacterial strains. Then, using the universal primers W001 (50-AGAGTTTGATCMTGGCTC-30) and W002 (50-GNTACCTTGTTACGACTT-30), all strains were identified by 16S rRNA sequencing. Following the manufacturer’s recommendations, amplicons were purified using the QIAquick PCR Purification Kit (QIAGEN GmbH, Hilden, Germany). Sequencing was performed by Macrogen (Hong Kong, China). Using the BLAST tool (http://www.ncbi.nih.gov/BLAST/, accessed on 20 April 2023), sequences were compared to those deposited in the GenBank database as well as to those in the Ribosomal Database Project (http://rdp.cme.msu.edu/edu/index.jsp, accessed on 20 April 2023).

### 2.3. Staphylococci Technological Characterization 

#### 2.3.1. Nitrate Reductase Activity 

The technique outlined by Miralles et al. [16] was used to assess nitrate reductase activity. After being resuspended in 50 mM phosphate buffer pH 7.0, cell pellets from overnight cultures were put into wells (6 mm in diameter) in Nutrient agar plates. Plates were flooded with 1 mL of a 1:1 solution of NIT1 (0.8 g of sulphanilic acid in 100 mL of 5 N acetic acid) and NIT 2 (0.6 g of N-Ndimethyl-1-Naphthylamine in 100 mL of 5 N acetic acid) for the detection of nitrite after 7, 24, and 72 h at 30, 20, and 15 °C, respectively.

#### 2.3.2. Proteolytic Activity 

Proteolytic activity was assessed on sarcoplasmic and myofibrillar proteins extracted according to Fadda et al. [17]. Briefly, lean ovine and pork meat were minced and diluted 1:10 (*w*/*v*) with 20 mmol/L phosphate buffer pH 6.5, homogenized in a StomacherLab-Blender400 (Seward Medical, London, UK) for 3 min, and centrifuged at 13,000× *g* for 20 min at 4 °C. The supernatant (sarcoplasmic fraction) was filter-sterilized (0.22 µm, Merck Millipore, Darmstadt, Germany) and stored, whilst the pellet (myofibrillar fraction) was resuspended in 200 mL of 0.03 mol/L phosphate buffer at pH 7.4 containing 0.1% (*v*/*v*) Triton X-100 and homogenized for 2 min in a Stomacher 400 blender. After centrifugation (13,000× *g* for 20 min at 4 °C), the pellet was washed three times with the same phosphate buffer to remove muscle proteases. The resulting pellet was weighed and resuspended in nine volumes of 0.1 mol/L phosphate buffer pH 6.5 containing 0.7 mol/L KI. After Stomacher homogenization for 8 min, the suspension was centrifuged (13,000× *g* for 20 min at 4 °C), and the supernatant was 10-fold diluted. Finally, the extract was filter sterilized (0.22 µm) and stored at 4 °C. The two protein fractions were quantified by Bio-rad Protein assay (Bio-rad, Segrate, Italy) and used in agar plate assay as well as for SDS-PAGE analysis.

For the agar plate assay, 50 µL of cell suspensions were loaded in 6 mm wells realized on PA medium (tryptone 5 g/L, yeast extract 2.5 g/L, glucose 1 g/L, agar 15 g/L, pH 6.9) supplemented with the protein extracts (0.5 mg/mL). After incubation for 48 h at 30 °C, plates were stained with Coomassie Blue R250 (0.1% in 40% methanol, 10% acetic acid) for 1 h and then water rinsed. Clear zones around wells indicated proteolytic activity [18].

Furthermore, cell suspensions washed three times with phosphate-buffered saline (PBS) were used to inoculate PA medium supplemented with 0.5 mg/mL protein extracts [19]. At time 0 and after 9, 18, and 27 days of incubation at 37 °C, cultures were centrifuged (5000 rpm for 15 min at 4 °C), and the supernatants were mixed with protein Laemmli buffer, heated at 95 °C for 5 minutes, and resolved by 12% SDS–polyacrylamide gel electrophoresis. The polypeptides’ molecular weight was estimated by the color-prestained protein standard (Biolabs, Pisa, Italy). Electrophoresis was carried out in a Mini Protean II (Bio-Rad) at 120 V. Gels were subsequently stained with Blue Coomassie for 1 h and then destained overnight.

#### 2.3.3. Lipolytic Activity

Cell suspensions in PBS (pH 7) were spotted onto Nutrient agar (Oxoid) plates with or without tributyrin (5%) added in place of Tween 80 (1% *v*/*v*). The development of a clear zone around the colony was indicative of lipase or esterase activity. Halos around the spots were daily measured after up to 5 days of incubation at 37 °C. Additionally, titration was used to determine lipolytic activity following the protocol proposed by Mauriello et al. [19]. After 7 days of incubation at 30 °C, broth cultures in YTF (1% tryptone, 0.5% yeast extract, 3% di NaCl, pH 7) added with pork fat (4% *w*/*v*) were subjected to lipid extraction using petroleum ether. The surface layer was then titrated with 0.1 N NaOH in ethanol using phenolphthalein (1%) as the indicator. Lipolytic activity, expressed as % of oleic acid, was evaluated according to the following equation: (a × N × 28.2)/g, where a is the amount (mL) of NaOH used for the titration, N is the normality, 28.2 is the % of the oleic acid equivalent weight, and g is the amount of pork fat in the sample.

#### 2.3.4. Antibiotic Resistance

Disk diffusion assay was performed on each isolate by following the M100 Clinical and Laboratory Standards Institute (CLSI) guidelines [20]. A total of ten antimicrobials applied on sterile disks were tested: chloramphenicol (30 μg), clindamycin (2 μg), penicillin G (10 U), amoxicillin (2 μg), erythromycin (15 μg), tetracycline (30 μg), ampicillin (10 μg), kanamycin (30 μg), gentamicin (10 μg), and vancomycin (30 μg). 

Inoculated Mueller–Hinton (Fisher Scientific, Horsham, UK) plates with antibiotic disks were incubated at 37 °C for 24 h. Inhibition zones were measured in millimeters using a ruler and interpreted using the guidelines and recommendations from CLSI M100 *Staphylococcus* spp. breakpoints [20], and guidelines were followed in cases when it was difficult to interpret zones of inhibition due to unclear edges [21]. 

#### 2.3.5. Biogenic Amines Production

The ability of the strains to form biogenic amines (BAs) was assessed by PCR as the presence of genes encoding for histidine (*hdc*), lysine (*ldc*), ornithine (*odc*), and tyrosine (*tdc*) decarboxylation. PCR conditions were those described by Yüceer and Özden Tuncer [22] and Landeta et al. [23]. *Enterococcus faecium* 65C1, isolated during a previous survey, was used as a positive control. 

## 3. Results and Discussion

### 3.1. Staphylococci Identification 

**Figure 1:**
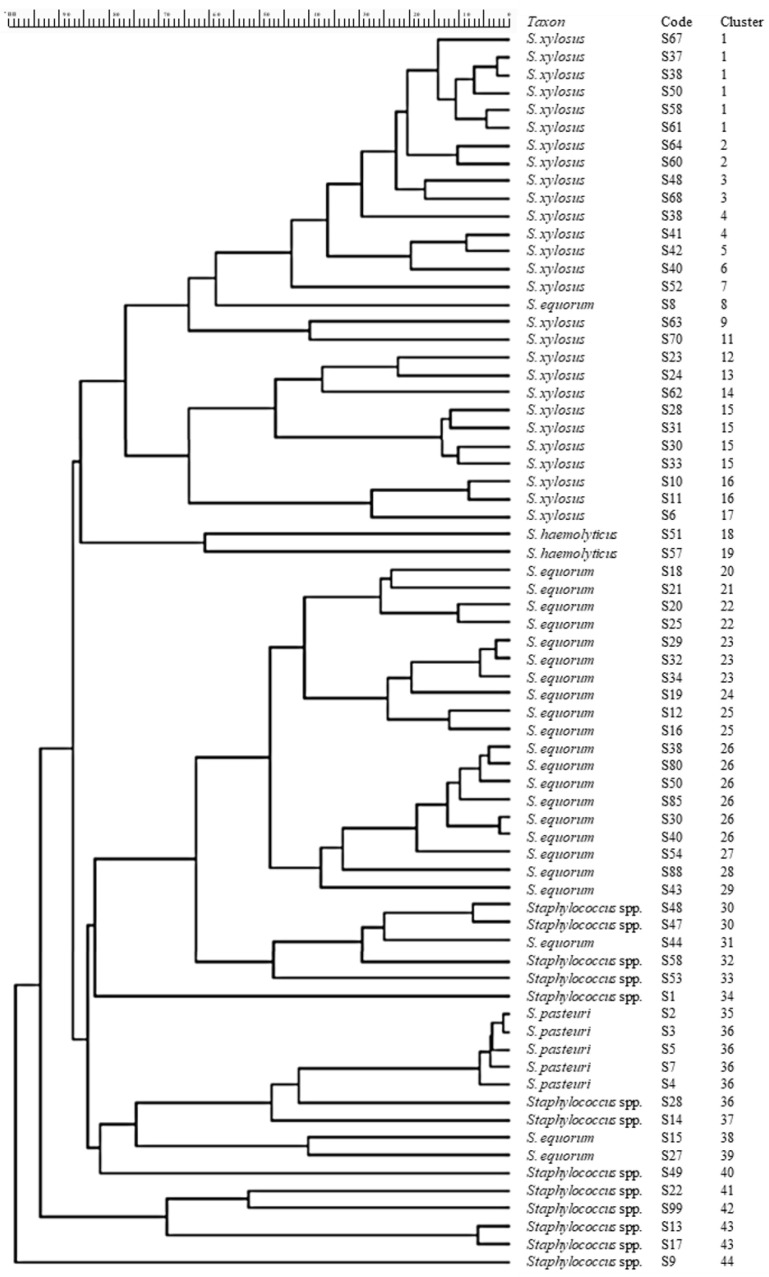
Dendrogram obtained by combining RAPD-PCR and rep-PCR fingerprints of 70 CoNS strains isolated from sheep sausages. Pearson’s correlation similarity coefficients were used to build a matrix of bacterial banding profiles’ similarities.

A total of 70 CoNS cultures were characterized by rep-PCR and M13-RAPD typing and identified by 16S rDNA sequencing. Data obtained with the two typing techniques were combined in a single dendrogram (Figure 1). By fixing at 85% of the similarity level, the 70 strains fell into 44 distinct clusters, 12 of which grouped more than one strain, and 32 strain-specific RAPD patterns. Following 16S rDNA sequencing, 27 isolates could be assigned to the species *S. xylosus* and 21 to *S. equorum,* which together accounted for more than 70% of the total isolates: 38.6% and 32.8%, respectively. Six isolates were reported to the species *S. pasteuri* (8.5%), two to *S. succinus* (2.8%), and two to *S. haemolyticus* (2.8%). For 12 strains, it was not possible to achieve a complete taxonomic collocation, and they were reported as *Staphylococcus* spp. (17.1%).

The prevalence of *S. xylosus* and *S. equorum* in Italian fermented sausages has already been reported by more than one author [19,24,25,26]. *S. saprophyticus* has been commonly isolated as well [14,25,27]. By contrast, the species *S. haemolyticus* is usually only seldom detected in meat-fermented products [19,28] but has been isolated from samples of pork, bovine, and sheep intestine sections used as casings [29].

### 3.2. Technological Characterization of Staphylococcus Strains

Biochemical tests for features of technological interest were performed on a pool of 14 selected cultures. Specifically, three strains of *S. xylosus* (S41, S48, and S63), five strains of *S. equorum* (S8, S29, S59, S60, and S65), and six *S. pasteuri* (S2, S3, S4, S5, S7, and S28) were assessed for proteolytic, lipolytic, and nitrate reductase activity, as well as for the presence of antibiotic resistance.

Two *S. xylosus* strains (S63 and S41) out of three exhibited nitrate reductase activity. Previous studies highlighted a variable nitrate reductase activity in this species. Sanchez Mainar and Leroy [30] reported that only three *S. xylosus* strains out of thirteen possessed high nitrate reductase activity, whilst, for Mauriello et al. [19], the occurrence of positive strains was higher: 57% of 23 analyzed strains. Similarly, all *S. capitis*, *S. pasteuri*, *S. xylosus*, and most of the *S. equorum* strains isolated from Llama sausages were able to reduce nitrate to nitrite, according to Rebecchi et al. [14], whereas none of the *S. pasteuri* and *S. equorum* strains here analyzed was able to express this trait.

None of the 14 strains was able to hydrolyze Tween 80. The ability to use tributyrin, on the other hand, was rather common (Table 1), and when it was evaluated by titration, the lipolytic capability was also confirmed on pork fat (Table 1). The strain *S. pasteuri* S3 showed the highest level of lipolytic activity and produced more oleic acid than 52%. Actually, the *S. pasteuri* strains S4 and S5 produced fairly high oleic acid levels: 4.51 and 4.31% of oleic acid, respectively. However, this species’ capacity for lipolysis has already been documented [31,32].

*S. xylosus* strains S41, S48, and S63 were able to hydrolyze pork fat, which resulted in significant amounts of oleic acid being produced: 3.91, 3.83, and 1.91%, respectively. Conversely, earlier research [19,33,34] revealed better lipolytic performances in strains of such species. Mangia et al. [3], in *S. xylosus* strains coming from pork sausages, detected higher levels of oleic acid liberation than those found for *S. pasteuri* in the current investigation.

All tested staphylococci did not show any activity against myofibrillar proteins, but nine out of fourteen were able to hydrolyze both pork and sheep sarcoplasmic proteins with halos that were always equal or greater than 1 cm (Table 1). Results were not consistent with those reported for Naples-type salami by Mauriello et al. [35], who claimed that *S. xylosus* and *S. equorum* species were more active on myofibrillar proteins than on sarcoplasmic ones.

**Figure 2:**
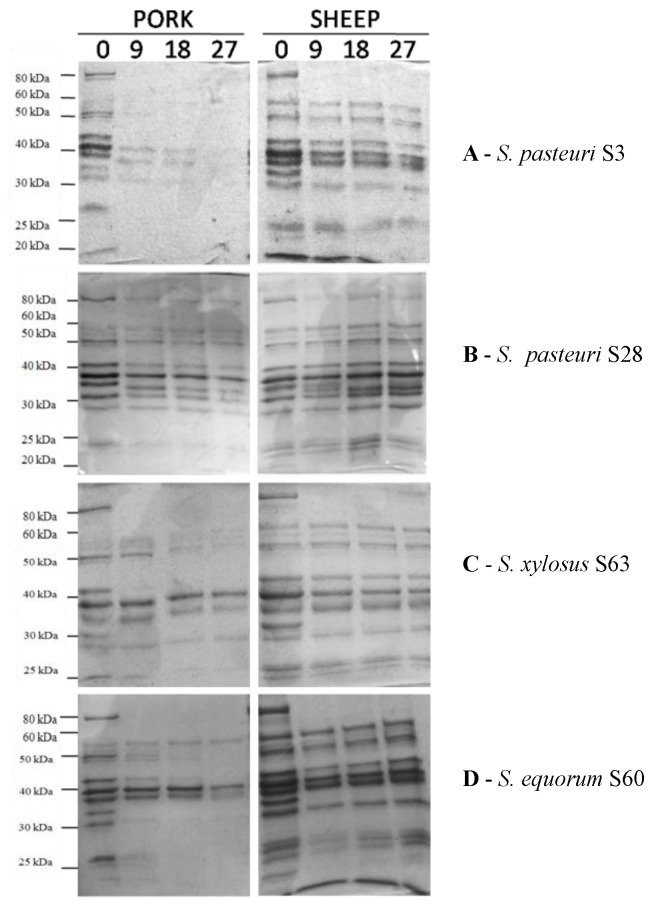
SDS-PAGE of protein (pork and ovine) extracts hydrolysis by selected CoNS at time 0 and after 9, 18, and 27 days of incubation at 37 °C.

SDS-PAGE was used to assess proteolysis as well. Within the first 9 days of incubation, the *S. pasteuri* S3 strain was able to completely hydrolyze all pork protein bands except those in the range of 30–50 kDa, which took 27 days of incubation (Figure 2A). Instead, there was no observed difference in the sheep sarcoplasmic proteins profile over the course of monitoring, except for bands at 80 and 32 kD that disappeared after 9 days. Except for a modest decrease in the strength of bands in the region of 30 to 25 kDa, profiles remained identical throughout monitoring (Figure 2A). Similar electrophoretic patterns were produced by strains S4, S5, and S7 of the same species that thus shared the *S. pasteuri* strain S3 proteolytic activity on sarcoplasmic proteins from sheep and pork. On the other hand, strain *S. pasteuri* S28 showed a completely different electrophoretic pattern (Figure 2B). Indeed, in pork substrate, at 9 days of incubation, the 38 kDa protein band disappeared, the 25 KDa band decreased in intensity, whilst an additional band at 35 kDa appeared. In sheep substrate, the appearance of two new bands at approximately 36 and 34 kDa after 9 days of incubation, coupled with the complete degradation of the band at 38 kDa after18–27 days and the partial hydrolysis of the 80 KDa band within 9 days, could be noticed as well.

In the *S. xylosus* S63 strain, the pattern of pork substrate proteolysis showed a partial degradation of the 25 kDa band within 9 days and of the 53 and 50 kDa protein fractions at 18 and 27 days of incubation; a complete degradation was observed for bands at 80, 42, and 32 kDa within the first 9 days of incubation. In the sheep substrate, bands at 80, 70, and 32 kDa were those that disappeared already after 9 days of incubation (Figure 2C).

The SDS-PAGE profile of *S. equorum* S60 represents all strains belonging to such species strains (S59, S60, S65). In pork, the complete hydrolysis of 80 and 32 KDa protein bands was observed at 9 days of incubation, whilst bands at 50, 48, 29, and 25 kDa were no longer visible after 18 days. Protein bands at 38 and 35 kDa underwent partial hydrolysis only after 27 days. In sheep substrate, bands at 28 and 25 kDa decreased in intensity at 9 days, whilst bands at 80, 32, and 20 kDa disappeared at the same time of incubation (Figure 2D).

The disappearance of pork sarcoplasmic proteins in the range of 50 kDa has already been reported [31,35]. On the other hand, no data are currently available on staphylococci proteolysis in sheep meat. It can only be assumed that the pH, as well as fat composition and presence in the sarcoplasmic reticulate, may explain the results. 

### 3.3. Strains’ Safety Assessment

The search for biogenic amine-encoding genes was conducted on 70 cultures. None of the strains tested by PCR was positive for the genes encoding histamine, cadaverine, putrescine, and tyramine production, except for the *S. pasteuri* S5 strain harboring the gene for tyramine. Actually, the production of BAs is not a widely distributed property among staphylococci isolated from dry-cured meat [23]. Recently, Van der Veken and coworkers [36] analyzed a total of 332 staphylococcal strains, mainly isolated from meat, highlighting a low incidence of BA production, with tyramine and β-phenylethylamine being the most common ones. 

The processing of meat products is a crucial factor in the spread of antibiotic resistance from farms, particularly from the manure to the environment, which includes animal food, water, and soil. Because of the global misuse or overuse of antibiotics in animal husbandry, microorganisms from farm environments and slaughterhouses, in particular, carry genes for antibiotic resistance. It has been shown that CoNS isolated from various habitats, foods, and countries carry one or more genes for antibiotic resistance [10]. 

Resistance to the primary antibiotics used for therapeutic purposes was relatively common in the current survey (Table 2).

All strains of *S. xylosus*, *S. equorum*, and *S. pasteuri* were amoxicillin- and kanamycin-resistant. *S. equorum* strains were sensitive to all other tested antibiotics. Actually, a low frequency of phenotypic antibiotic resistance is frequently observed in this species [10,37,38]. *S. xylosus* strains were all resistant to penicillin B. Marty et al. [38] detected a high resistance to penicillin in *S. xylosus* isolates originating from spontaneously fermented meat products. By contrast, Fontana et al. [39] indicated *S. xylosus* as the most tetracycline-resistant species, while, in this case, none of the analyzed strains possessed this feature. Conversely, all *S. pasteuri* strains were resistant to both ampicillin and penicillin B, and four out of five strains exhibited tetracycline resistance. 

Regarding vancomycin resistance, according to the CLSI [20] standard, the disk diffusion method did not provide a precise separation between sensitive and resistant organisms. 

The generally identical behavior of isolates within the same species confirmed that antibiotic resistance is species-dependent [37].

The detection of multiple antibiotic resistances is not unexpected, given the context of spontaneously fermented meat products. However, antibiotic resistance is hosted by strains isolated from spontaneously fermented meat products exclusively made by using meat from wildlife or animals grown under low- or no-antibiotic pressure [39].

## 4. Conclusions

Sausage manufacture by using sheep or goat meat is quite widespread in several regions apart from Sardinia. Many of these products correspond to ancient methods of preserving meat at a time when there was no other way of processing than curing with salts, air drying, or smoking. In the present study, the CoNS community associated with sheep sausages produced in Sardinia was evaluated for the very first time. The high variability in the production and the coliforms occurrence make the search for adjunct cultures of crucial relevance. In this perspective, the characterization of the autochthonous CSN population represents a first step for approaching a starter selection. Selected strains of *S. pasteuri*, such as S3 and S4, showing low antibiotic resistance but good proteolytic and lipolytic activity and are negative for genes encoding enzymes that produce BAs, could be eligible candidates for the formulation of autochthonous starter cultures in combination with Lactic Acid Bacteria strains. Of course, more research needs to be conducted to track the effectiveness of the selected cultures in meat models and experimental processing of sausages.

## Figures and Tables

**Table 1:** Lipolytic and proteolytic activity of 14 selected CoNS. Analyses were performed in triplicate. Results are reported as mean values ± SD.

Taxon	Strain Code	Lipolysis		Proteolysis on Sarcoplasmic Proteins	
		Tributyrin ^a^	% Oleic Acid ^b^	Pork ^c^	Sheep
*S. xylosus*	S41	1.4 ± 0.10	3.91 ± 0.85	-	-
	S48	1.25 ± 0.05	3.83 ± 0.00	-	-
	S63	1.95 ± 0.04	1.91 ± 0.06	1.2	1.05
*S. equorum*	S8	1.29 ± 0.07	3.22 ± 0.01	-	-
	S29	1.30 ± 0.01	2.90 ± 0.24	-	-
	S59	-	-	1.1	1.10
	S60	1.63 ± 0.18	1.69 ± 0.04	1.2	1.00
	S65	-	-	1.1	1.10
*S. pasteuri*	S2	1.55 ± 0.05	3.93 ± 0.70	-	-
	S3	1.40 ± 0.03	5.02 ± 0.18	1.0	1.00
	S4	1.45 ± 0.02	4.51 ± 0.32	1.0	1.00
	S5	1.37 ± 0.50	4.31 ± 0.16	1.0	1.00
	S7	1.58 ± 0.03	3.12 ± 0.10	0.9	0.85
	S28	-	-	1.1	1.10

^a^ Diameter (cm) of halos surrounding colonies on Nutrient agar enriched with tributyrin. ^b^ Oleic acid equivalents assessed by the titration method. ^c^ Diameter (cm) of the clear halos around wells.

**Table 2:** Antibiotic susceptibility of *Staphylococcus* strains.

Taxon	Strains	Antibiotics									
		Gen ^1^	Kan	Tet	Clo	Amp	Van	Ery	Amx	Pen	Cly
*S. xylosus*	S41	S ^2^	R	S	S	S	ND	S	R	R	S
	S48	S	R	S	S	S	ND	S	R	R	S
	S63	S	R	S	S	S	ND	S	R	R	S
	S69	S	R	S	S	S	ND	S	R	R	S
*S. pasteuri*	S2	S	R	R	S	R	ND	S	R	R	S
	S3	S	R	R	S	R	ND	S	R	R	S
	S4	S	R	R	S	R	ND	S	R	R	S
	S5	S	R	R	S	R	ND	S	R	R	S
	S7	S	R	I	S	R	ND	S	R	R	S
*S. equorum*	S8	S	R	S	S	S	ND	S	R	S	S
	S29	S	R	S	S	S	ND	S	R	S	S
	S59	S	R	S	S	S	ND	S	R	S	S
	S60	S	R	S	S	S	ND	S	R	S	S
	S65	S	R	S	S	S	ND	S	R	S	S

^1^ Antibiotic resistance (Gen) gentamicin; (Kan) kanamycin; (Tet) tetracycline; (Clo) chloramphenicol; (Amp) ampicillin; (Van) vancomycin; (Ery) erythromycin; (Amx) Amoxicillin; (Cli) clindamycin; (Pen) penicillin B. ^2^ R: resistant; I: intermedium resistance; S: sensitive. Disc diffusion susceptibility testing was performed according to the 2022 CLSI 32nd ed. (CLSI supplement M100) [20] and EUCAST v.12.0 guidelines [21].

## Data Availability

The original contributions presented in the study are included in the article, further inquiries can be directed to the corresponding author.

## Supplementary Materials

The following supporting information can be downloaded at: https://www.mdpi.com/article/10.3390/foods13040633/s1, Figure S1: Flow chart of the sheep sausages manufacturing process.
