# Supplementary figures and images for: Technological and Safety Characterization of Coagulase-Negative Staphylococci Isolated from Sardinian Fermented Sausage Made by Ovine Meat
Source: Foods. 2024 Feb 19;13(4):633. doi: 10.3390/foods13040633 (PMC10888299; doi:10.3390/foods13040633)

## Supplementary Figure S1

Flow chart of the sheep sausages manufacturing process

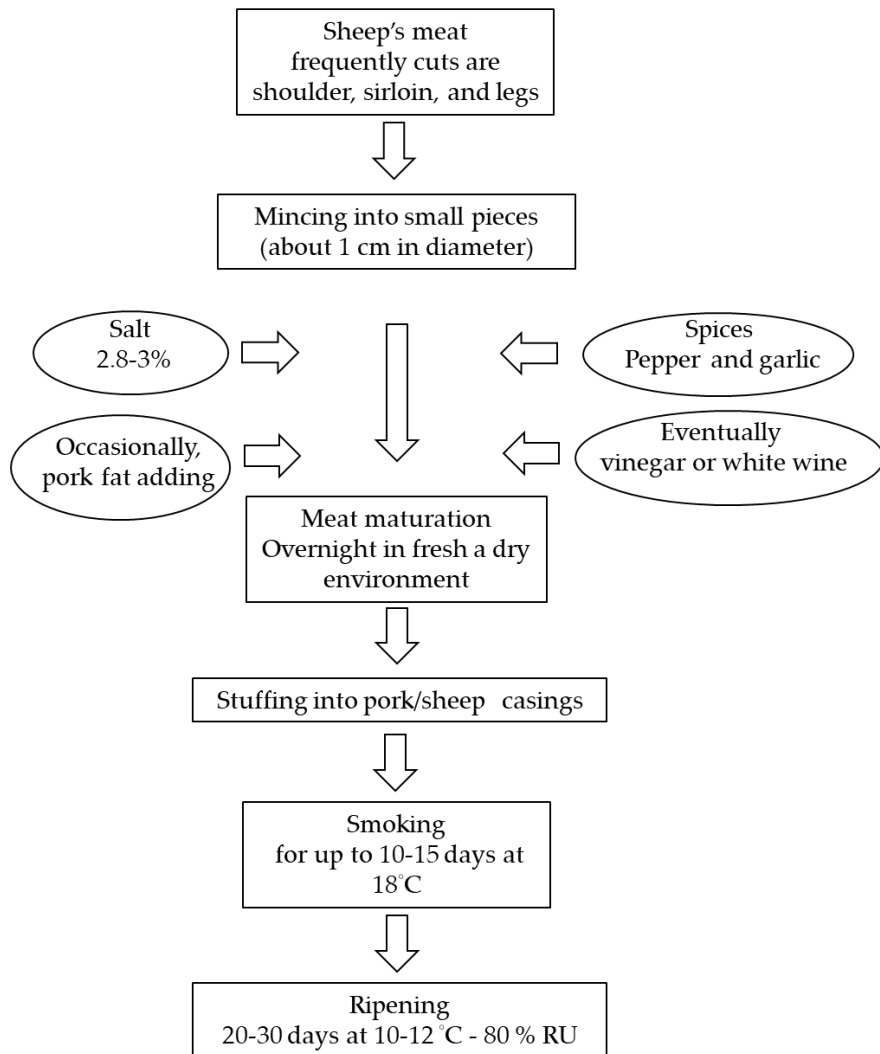

Supplement: Supplementary file 1 [file foods-13-00633-s001.zip › foods-2850110-supplementary.pdf]
